# Proactive Fault Diagnosis of a Radiator: A Combination of Gaussian Mixture Model and LSTM Autoencoder

**DOI:** 10.3390/s23218688

**Published:** 2023-10-24

**Authors:** Jeong-Geun Lee, Deok-Hwan Kim, Jang Hyun Lee

**Affiliations:** 1 Department of Smart Digital Engineering, INHA University, Incheon 22212, Republic of Korea; jeonggeun2.lee@doosan.com; 2 Doosan Industrial Vehicle Co., Ltd., Incheon 22503, Republic of Korea; 3 Department of Electronic Engineering, INHA University, Incheon 22212, Republic of Korea; deokhwan@inha.ac.kr; 4 Department of Naval Architecture and Ocean Engineering, INHA University, Incheon 22212, Republic of Korea

**Keywords:** PHM, radiator, vibration, anomaly detection, machine learning, PCA, deep learning, LSTM autoencoder, GMM

## Abstract

Radiator reliability is crucial in environments characterized by high temperatures and friction, where prompt interventions are often required to prevent system failures. This study introduces a proactive approach to radiator fault diagnosis, leveraging the integration of the Gaussian Mixture Model and Long-Short Term Memory autoencoders. Vibration signals from radiators were systematically collected through randomized durability vibration bench tests, resulting in four operating states—two normal, one unknown, and one faulty. Time-domain statistical features of these signals were extracted and subjected to Principal Component Analysis to facilitate efficient data interpretation. Subsequently, this study discusses the comparative effectiveness of the Gaussian Mixture Model and Long Short-Term Memory in fault detection. Gaussian Mixture Models are deployed for initial fault classification, leveraging their clustering capabilities, while Long-Short Term Memory autoencoders excel in capturing time-dependent sequences, facilitating advanced anomaly detection for previously unencountered faults. This alignment offers a potent and adaptable solution for radiator fault diagnosis, particularly in challenging high-temperature or high-friction environments. Consequently, the proposed methodology not only provides a robust framework for early-stage fault diagnosis but also effectively balances diagnostic capabilities during operation. Additionally, this study presents the foundation for advancing reliability life assessment in accelerated life testing, achieved through dynamic threshold adjustments using both the absolute log-likelihood distribution of the Gaussian Mixture Model and the reconstruction error distribution of the Long-Short Term Memory autoencoder model.

## 1. Introduction

This study discusses the application of Prognostics and Health Management (PHM) techniques [1,2,3] to prevent radiator failures, enhance equipment availability, and consequently reduce maintenance costs. The primary objective of PHM is to proactively detect early indicators of radiator malfunctions. In facilities and mechanical equipment powered by internal combustion engines, radiators are pivotal in preventing overheating. However, when operating in demanding environments, radiators face significant challenges. These include temperature fluctuations and repetitive loads transferred from the machinery that supports the radiators. Under high-temperature or high-friction conditions, radiators can experience temperature spikes that exceed critical thresholds. Additionally, mechanical damage may arise when a radiator’s natural frequency resonates with the vibrations present in the facility where it is installed. Such resonant vibrations can potentially induce cumulative fatigue failures in the welded joints of the radiator’s tubes.

Based on the experience of conducting accelerated reliability experiments on radiators in a laboratory environment, it is necessary to analyze the progression of faults in real operational conditions and proactively detect failures [4]. Nevertheless, it is challenging to determine the exact point of failure during the reliability testing process solely through visual observation or time-series changes in sensor data. Therefore, a precise method for diagnosing failures during the reliability testing process is required. Using machine learning classification, interpolation algorithms, or deep learning can enhance fault detection and the accuracy of fault determination criteria in reliability experiments. This allows for proactive fault detection by observing the system’s condition in real operational environments [5]. There are several approaches to fault diagnosis, including data-driven, model-based, and knowledge-based methods. Lan et al. established electrical and thermal models for lithium-ion batteries and proposed a model-based fault diagnosis system for battery sensors [6]. Model-based diagnostic techniques require complex physical models or governing equations of the target system and verification through simulation. In contrast, sensor data-driven fault diagnosis can distinguish abnormal behavior from signals that represent the system’s state [1]. Various machine learning algorithms, such as classification, prediction, clustering, and time series forecasting, can be applied to fault diagnosis. However, it is necessary to extract features that align with the characteristics of the equipment and fault indicators in order to develop fault detection algorithms [2]. Sayyad et al. demonstrated that by applying signal processing, feature engineering, Convolution Neural Network (CNN), and Long Short-Term Memory (LSTM) to the vibration signals of tool wear in milling machines from the IEEE NUAA Ideahous dataset [7], it is possible to predict the remaining useful lifetime (RUL) [8]. However, even when data are collected from equipment under real operating conditions, it can be difficult to accurately label normal and faulty states. Therefore, the use of unsupervised learning-based fault diagnosis techniques, such as clustering and anomaly detection, becomes necessary [5]. Machine learning has significantly advanced the field of fault diagnosis by offering a range of algorithms capable of identifying abnormal conditions with high accuracy [9]. Among various machine learning techniques, Gaussian Mixture Models (GMMs) are frequently used for clustering and density estimation tasks. These models have found applications in fault diagnosis to model normal behavior, thus aiding in the identification of anomalies [10]. However, GMMs have limitations, such as their inability to capture temporal dependencies, making them less suitable for tasks requiring sequence-based learning [11]. In contrast, Long Short-Term Memory networks, a type of recurrent neural network, excel in learning from sequences. These networks have shown promise in fault diagnosis and anomaly detection in time-series data [12]. Nonetheless, LSTM is computationally intensive and is sensitive to hyperparameter settings [13,14]. In industrial settings, particularly in the context of radiator fault diagnosis, anomaly detection is a critical tool for proactive maintenance [15]. Existing methods often involve extensive manual feature extraction, making them less adaptive to new, unseen anomalies [16]. These methods primarily suffer from an inability to adapt to new types of faults and insensitivity to early-stage anomalies. The requirement for manual labor in feature extraction remains a significant drawback [17].

**Figure 1:**
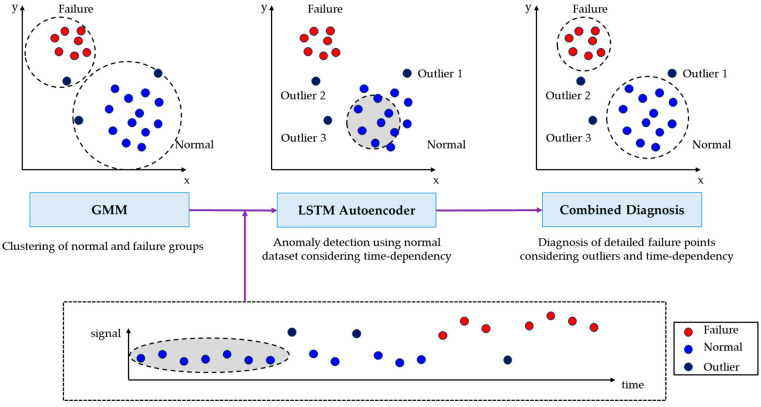
Fault diagnosis procedure integrated with GMM and LSTM Autoencoder.

In light of the current advancements and identified limitations of LSTM as discussed based on the above literature, this study aims to enhance the fault diagnosis process for radiators and related equipment. The approach chosen in this study combines GMM and LSTM to compensate for the shortcomings of LSTM and aims to balance the methods. This combination is tailored to address the challenges associated with diagnosing radiator faults in high-temperature or high-friction environments. Consequently, this study aims to improve equipment availability, prevent catastrophic failures, and significantly reduce maintenance costs. In doing so, this study fills existing technological gaps by adopting a combination approach that leverages GMMs for initial classification and LSTMs for sequence-based learning. GMMs are chosen for their strong clustering capabilities, useful for diagnosing known types of faults, while LSTMs are employed to capture the temporal sequences in data, thereby offering advanced anomaly detection for unknown or new types of radiator faults. In this study, both unsupervised learning models, GMM and LSTM autoencoder, were employed for radiator fault diagnosis. The choice of these two particular machine learning models is motivated by their complementary strengths: GMM excels at initial fault classification based on its clustering capabilities, while LSTM specializes in capturing time-dependent sequences, allowing for the advanced detection of unknown or new types of radiator fault. Figure 1 summarizes the procedure for the combined diagnosis of GMM and LSTM. Initially, GMM is employed to classify normal and abnormal cases, including outliers. Subsequently, considering time-series characteristics, the LSTM autoencoder is used to train only on normal data, enabling the classification of normal and faulty states based on the progression over time. Furthermore, one of the strengths of time-series analysis is that it does not solely diagnose normal and faulty states based on the condition at each data point but also considers the states of the data before and after each point. This combined diagnosis enhances the accuracy of fault diagnosis and allows for precise fault detection, thereby improving the accuracy of reliability testing. In addition, this approach allows analysis of outlier, normal and fault conditions over time in real-world operating environments, not just limited to reliability testing. Such methods enable detailed fault diagnosis even in scenarios where labels are difficult to obtain, both in reliability testing and in real-world applications. 

In the subsequent sections, a comprehensive breakdown of the proposed methodology unfolds, commencing with:

- Section 2—Analyzing the Effects of Vibration on Radiator Integrity: In this section, an in-depth analysis of frequency response signals obtained from a real operational facility is undertaken to simulate the impact of vibrations on the structural integrity of radiators. A randomized durability vibration bench test is conducted, employing state-of-the-art acceleration sensors for real-time signal capture. The rationale behind the selection of GMM and LSTM autoencoders for fault diagnosis is elaborated upon.
- Section 3—Feature Engineering and Data Refinement: This section is dedicated to the critical task of feature engineering. Here, the focus is on extracting time-domain statistical features from raw data and refining them through Principal Component Analysis (PCA). The insights gained from PCA guide the subsequent training of the GMM. Findings related to fault diagnosis for stages 2 and 4, along with an in-depth exploration of anomaly detection for the unlabeled stage 3, are presented.
- Section 4—Leveraging LSTM Autoencoders: In this section, attention turns to the practical implementation of LSTM autoencoders for fault diagnosis and anomaly detection. The advantages of this approach are highlighted, showcasing its superiority over traditional methods, especially when dealing with time-series data.
- Section 5—Evaluation and Technical Contributions: The final section provides a comprehensive evaluation of the proposed combination of GMM and LSTM autoencoders. Here, meticulous detailing of the technical contributions of the methodology to the field of machine learning for fault diagnosis is presented. Emphasis is placed on its capability for precise fault detection and anomaly prediction. The practical implications of this approach for real-world applications, such as enhancing equipment uptime, preventing critical failures, and minimizing maintenance costs, are explored.

By integrating the strengths of GMM and LSTM within a unified framework, this study addresses the existing technological gaps in radiator fault diagnosis. The approach stands as a robust, adaptable, and highly efficient solution poised to make significant contributions in the domain.

## 2. Radiator Dataset and Research Objective

### 2.1. Random Durability Vibration Bench Test and Dataset

**Figure 2:**
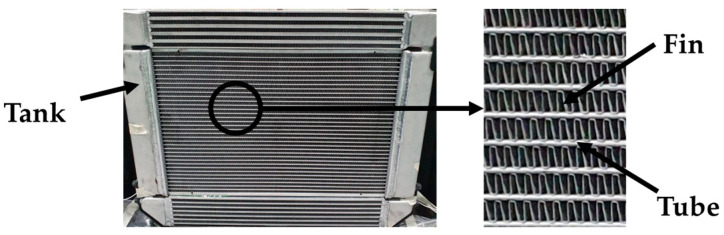
Radiator components: tank, fin, and tube.

The focus of this study is on fin-and-tube heat exchangers, commonly known as radiators. Specifically, the study centers on cross-flow heat exchangers in which coolant circulates laterally, either from left to right or vice versa. Such exchangers feature tanks situated on both ends, interconnected by tubes that facilitate coolant flow, as depicted in Figure 2. A cooling fan aids in dissipating waste heat from these tubes into the ambient environment. Moreover, to increase the heat transfer surface area and thereby enhance cooling efficiency, fins are strategically placed between the tubes [18].

The primary material comprising the tank-tube-fin assembly is an aluminum alloy, well-known for its excellent thermal conductivity and efficient cooling properties. Aluminum brazing techniques are used to securely join these individual components. Notably, radiator welds are susceptible to breakage, often due to equipment-induced vibrations. Such failures result in coolant leakage, leading to operational downtime and increased maintenance expenses.

Given the identified vulnerability of radiator welds and the resulting faults, this study conducted two targeted experiments to simulate and promptly address radiator degradation. Initially, the vibration signal from the equipment impacting the radiator was captured. This captured signal served as the load profile for a random durability vibration bench test. The Accelerated Life Test (ALT) method was employed, aiming to artificially hasten the life degradation of test specimens to bolster lifetime reliability predictions and to minimize test durations and sample requirements [19,20,21,22,23]. Specifically, ALT introduces more extreme conditions than those usually encountered, thereby shortening required test time and sample counts [19,20,21,22,23]. Random durability vibration tests are especially suited for ALT, as they allow for the simultaneous introduction of multiple sinusoidal vibration components, closely mimicking real-world conditions.

To further clarify the relevance of these experiments, it is important to understand that radiator failures generally manifest in one of two ways: either the radiator’s natural frequency comes into resonance with the vibrations from the equipment, or failures occur at the material tube welds, where fatigue loads tend to accumulate. In line with this, vibration signals from the radiator were specifically gathered during bench tests to simulate and accelerate fatigue failure scenarios at these tube welds. 

**Figure 3:**
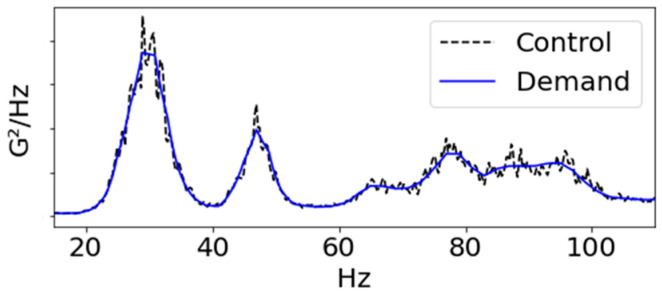
Y-axis PSD profile: comparison of demand and control settings for random durability vibration bench test.

**Figure 4:**
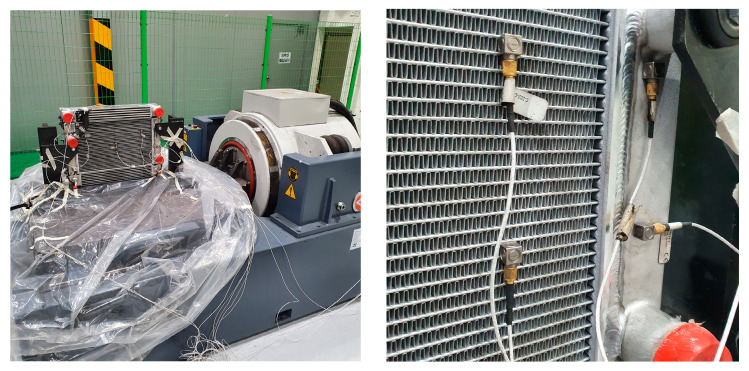
Experimental setup: radiator equipped with acceleration sensors mounted on a vibration dynamo.

By analyzing the Power Spectral Density (PSD) of the vibration signal applied to the radiator, it was determined that fatigue failure of the radiator primarily occurred in the Y-axis direction. Consequently, a random durability vibration test was conducted, using the Y-axis PSD as the load profile (as shown in Figure 3 and Figure 4). PSD is a technique employed for analyzing the outcomes of a Fast Fourier Transform (FFT) on an acceleration response signal. In this process, the FFT output (G) is multiplied by a complex number component, generating real-number amplitudes (G^2^). These amplitudes are then divided by the frequency resolution to produce a shape. Importantly, PSD is independent of the frequency resolution, making it advantageous for comparing vibration levels across signals. Due to these merits, PSD is often utilized to describe vibration characteristics in various random vibration specifications. Noteworthy standards for sinusoidal vibration testing include IEC 60068-2-6 [24] and the U.S. military specification MIL-STD-810 [25].

**Figure 5:**
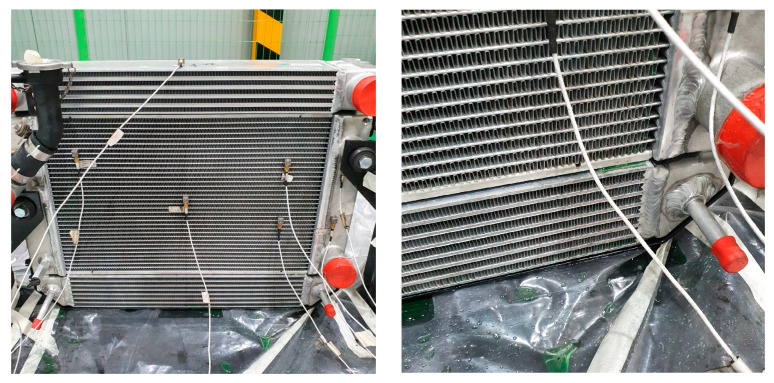
Coolant leakage observed during radiator durability test.

During the random durability vibration test, eight acceleration sensors were affixed to the radiator to capture signals at a sampling rate of 12,800 Hz over the course of the test, which lasted 808 min. For the first 300 min, the radiator showed no signs of damage to the radiator. However, around the 400-min mark, a fracture appeared in a weld on the radiator tube, causing a minor coolant leak. By the end of the test, the damage to the radiator had progressed significantly, leading to a major coolant leak, as depicted in Figure 5. The data collected from this test has been labeled and are provided in Table 1 for further reference.

During the experimental process, signals were sampled at an elevated rate of 12,800 Hz. This ensured the detection of even the most subtle changes in the system’s condition, which is pivotal for effective anomaly detection (refer to Table 2). By down-sampling the reliability test data with high-resolution, we ensured the applicability of fault diagnosis algorithms even to low-resolution data typically collected during operational processes. This strategic adjustment enabled us to harmonize the sampling rate of lab-based data with that of real-world scenarios.

Table 2 provides a comprehensive breakdown of the equipment utilized for data acquisition, detailing sensor specifications and data types. Data was procured using eight tri-axial acceleration sensors, integrated with Siemens SCADAS equipment. It is worth noting that this study was predominantly concerned with identifying faults associated with y-axis vibration acceleration. Consequently, only data pertaining to the y-axis was harnessed, yielding a total of eight distinct acceleration signals.

### 2.2. Methods and Evluation Metrics

Numerous approaches have been proposed in various studies to diagnose faults, with classifiers ranging from Neural Networks, Logistic Regression, Support Vector Machines (SVM), Random Forest, XGBoost, to LightGBM [26,27,28,29,30,31]. Traditional classifiers, as mentioned above, predominantly operate within the supervised learning paradigm. This necessitates the availability of pre-classified abnormal data accompanied by the respective labels for effective training. This necessitates the availability of pre-classified abnormal data accompanied by the respective labels for effective training. Obtaining such datasets from operational radiators in actual working conditions often poses considerable challenges. Furthermore, these classifiers face challenges, particularly when faced with newly emerging failure types that have not been part of their training regimen. To address these inherent challenges, this study employs unsupervised learning techniques. Specifically, we have chosen Gaussian Mixture Models [32,33] and LSTM autoencoders [34,35] for classifying data in stages 2 and 4. Notably, these models have demonstrated proficiency in diagnosing faults during stage 3, the attributes of which remain relatively elusive. When applied to real-world equipment, these models have the potential to detect both hidden and known failure modes, thereby improving diagnostic capabilities. Over time, the integration of these new failure cases and labels with existing supervised classifiers and expert knowledge is expected to enable more accurate and comprehensive fault diagnosis.

The performance of the chosen unsupervised models is evaluated using the Area Under the Curve (AUC) metric. This metric is particularly useful when data do not have clear distinctions between normal and abnormal conditions, and where precise threshold selection is challenging. The AUC quantifies the performance of a model without requiring a specific threshold and is calculated based on the Receiver Operating Characteristic (ROC) curve. This curve illustrates the relationship between the True Positive Rate (TPR) and the False Positive Rate (FPR). A higher AUC score, ranging between 0 and 1, indicates better model performance. Specifically, a perfect classifier would achieve an AUC close to 1, while a random classifier would score around 0.5. Any performance falling below this threshold value, resulting in an AUC of less than 0.5, would be considered worse than random classification. The AUC score serves as the evaluation metric for the binary classification results of stages 2 and 4, based on the thresholds established during stage 1. Detailed diagnosis of unknown conditions in stage 3 is conducted using the thresholds of the trained unsupervised models.

## 3. GMM-Based Fault Diagnosis

### 3.1. Feature Engineering and PCA of Radiator Dataset

**Figure 6:**
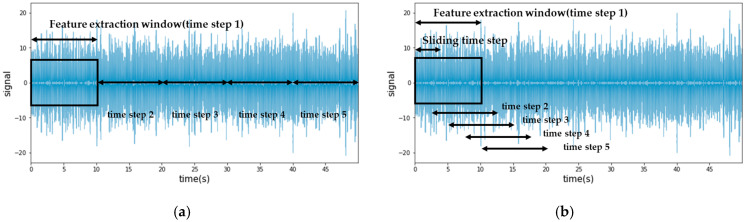
Comparison of feature extraction from time series: (**a**) Conventional method; (**b**) Sliding window augmentation method.

The dataset included eight acceleration signals, with each time series signal containing 620,544,000 data points, collected at a rate of 12,800 Hz for 808 min. Given the large volume of data, which could impede efficient analysis, feature engineering techniques were employed for data compression. Specifically, seven statistical features—minimum, maximum, absolute mean, variance, root mean square (RMS), skewness, and kurtosis—were extracted for each time window, ranging from one to ten seconds. This yielded 56 feature vectors, effectively transforming the data dimensions from 620,544,000 × 8 × 1 to 4848 × 8 × 7 in a ten-second window. It should be noted that increasing the window size reduces the number of feature vectors. To augment the feature vectors and ensure sufficient training data, a sliding window technique was applied, as illustrated in Figure 6, which depicts the difference between the conventional and sliding window augmentation methods. This technique increased the number of ten-second feature vectors from 4,848 to 48,471.

**Figure 7:**
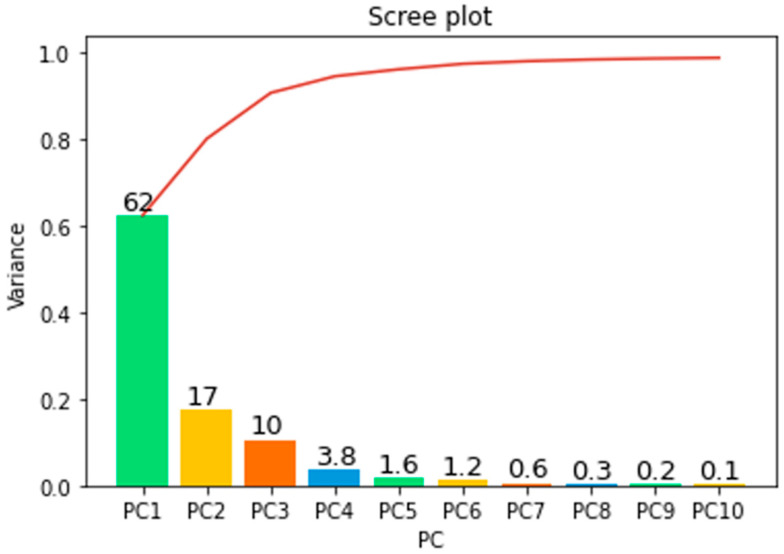
Scree plot illustrating PCA results for the radiator feature dataset.

Feature vectors derived from ten-second windows using a sliding window technique underwent dimensionality reduction through PCA. This led to observable state changes in the data. PCA serves as a method to minimize data dimensionality by generating new variables, known as principal components, which encapsulate the key information of the original dataset [36]. A scree plot, along with a list of eigenvalues of these principal components in descending order, was analyzed to determine the optimal number of components for data representation (shown in Figure 7). The scree plot serves as a vital tool for assessing the impact of PCA on the dataset and indicating the appropriate number of principal components [37]. In this study, the first principal component was identified as a linear combination of variables accounting for the maximum variance, whereas the second principal component, geometrically orthogonal to the first, explained the next highest variance. The analysis indicated that the first and second principal components collectively accounted for 62% and 17% of the data variance, respectively, explaining 89% of the total variance.

**Figure 8:**
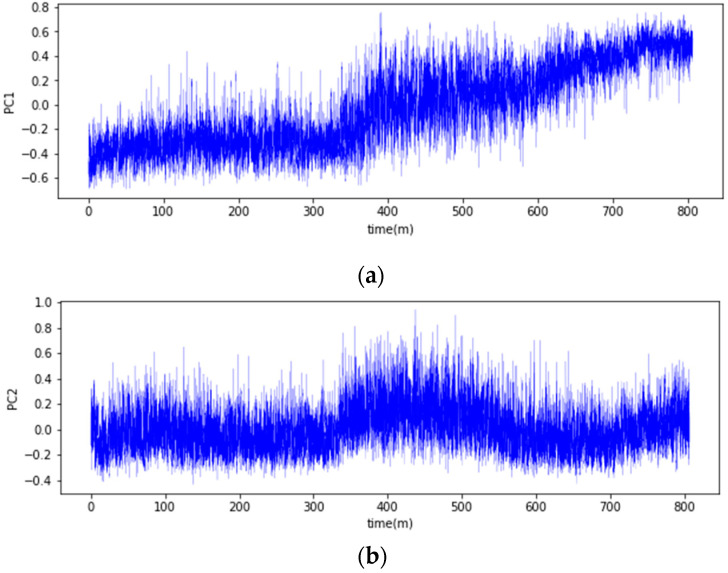
Temporal changes of principal components: (**a**) PC1; (**b**) PC2.

As depicted in Figure 8, a time-series graph visualizing the first and second principal components (PC1 and PC2) was generated. The graph showed a gradual temporal rise in the values of PC1, but this increase was accompanied by notable fluctuations, making it challenging to accurately discern state changes. Likewise, PC2 also displayed substantial fluctuations, further complicating the visual differentiation between normal and abnormal data distributions.

**Figure 9:**
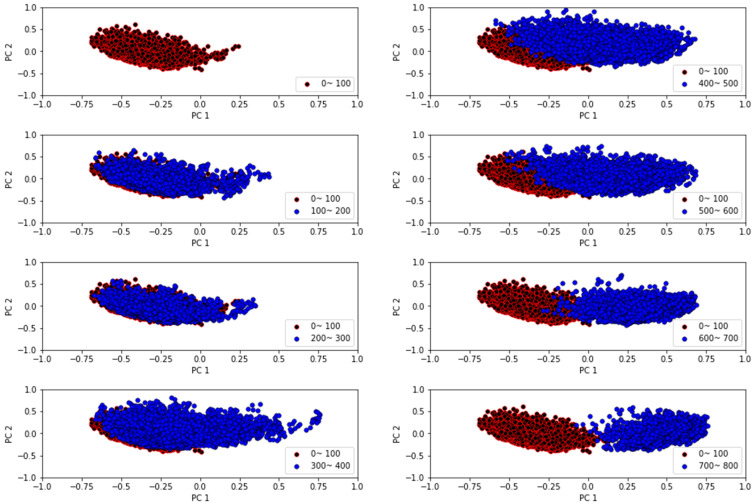
Scatter plot of principle components over time.

Building on the limitations of the time-series graph discussed in Figure 8, a two-dimensional scatter plot was constructed to further investigate the distribution of PC1 and PC2 values. As illustrated in Figure 9, this visualization revealed changes in the cluster’s state when compared to the normal state observed during stage 1 (0–100 min). However, data from stage 3 (300–400 min), where radiator failure was anticipated, showed considerable overlap with stage 1. A similar overlap was evident with data up to 600 min in stage 4. Nonetheless, the data from the end-failure portion of the experiment exhibited distinct characteristics compared to the normal state data.

In light of the challenges highlighted in the time-series visualization in Figure 8, a two-dimensional scatter plot of PC1 and PC2 was constructed, as depicted in Figure 9. This visualization facilitated a more detailed observation of cluster state changes relative to the normal state observed during stage 1 (0–100 min). However, the data from stage 3 (300–400 min), where radiator failure was anticipated, exhibited significant overlap with stage 1. A comparable overlap was evident with data up to 600 min in stage 4. Despite these overlaps, the data corresponding to the end-failure part of the experiment displayed distinct characteristics compared to the normal state data.

**Figure 10:**
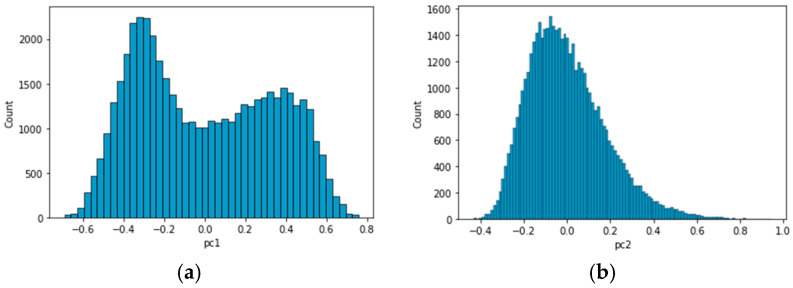
Histograms of principal components: (**a**) PC1; (**b**) PC2.

Given the intricacies highlighted in Figure 8 and Figure 9, an analysis of the histograms of principal components 1 and 2 was undertaken, as shown in Figure 10. This analysis revealed that PC1 displayed a bimodal distribution, characterized by two distinct peaks. In contrast, PC2 presented an unimodal distribution, distinguished by a single peak. Typically, a bimodal distribution indicates the presence of two different underlying distributions, each with its own set of characteristics or mean values. In this case, the bimodal nature of PC1 is believed to represent a mixture of data from both normal and abnormal states.

### 3.2. Fault Diagnosis and Anomaly Detection Result Using GMM

Building on the histogram analysis that identified a bimodal distribution for PC1, this section investigates the application of Gaussian Mixture Models for classifying normal and abnormal data. Recognized as an unsupervised learning technique, GMM employs probabilistic modeling to depict complex datasets as combinations of simpler probability distributions. Specifically, the GMM framework represents data as an ensemble of multiple Gaussian distributions, inferring that the data emerges from several clusters. For each of these clusters, the GMM computes the mean and covariance matrix of the Gaussian distributions. Data points are then attributed to clusters based on the probability of each data point belonging to a particular cluster. By determining the probability for each cluster across all data points, assignments are made to the cluster exhibiting the highest probability. This versatility allows GMM to function proficiently even in scenarios where distributions intersect or overlap. Thus, even if the dataset encompasses multiple diverse Gaussian distributions, GMM can aptly model the data. 

To optimize the GMM, the Expectation-Maximization (EM) algorithm is employed to minimize the negative log-likelihood during the learning process, as cited in [38]. In the context of GMM, the EM algorithm estimates the model parameters, specifically the mean and covariance of each Gaussian distribution that constitutes the model. The log-likelihood serves as an indicator of how well the model fits the data. A higher log-likelihood value signifies a better fit of the model to the data. GMM aims to minimize the negative value of the log-likelihood during the training phase. This approach is commonly taken in optimization problems because minimization is often computationally more tractable than maximization. Consequently, a higher absolute value of the negative log-likelihood would suggest that the data point is not well-represented by the model. Given a dataset D and model parameters Θ, the *log − likelihood* is expressed as follows [32]:

(1) $$log-likelihood=log\;p\left(D|\Theta \right)$$

Two or more Gaussian distributions were determined based on the analysis results of PC1. The GMM was trained using the overall data of PC1 and PC2 from stages 1 to 4. Before initiating fault diagnosis, the number of Gaussian components in the GMM was carefully evaluated by varying the component count from 1 to 5. The Bayesian Information Criterion (*BIC*) and Akaike Information Criterion (*AIC*) were employed to assess the model fit for each distribution. An inappropriate selection in the number of Gaussians can lead to model overfitting, thus compromising its reliability and predictive power. *BIC* and *AIC* serve as pivotal metrics in model selection, balancing fit and complexity, as cited in [39]. *BIC* is particularly tailored to account for the uncertainties and is defined as follows [39]:

(2) BIC=−2⋅log−likelihood+k⋅log(n)

where, k represents the number of parameters in the model. In the context of GMM, the mean and covariance are parameters for each Gaussian distribution, so the value of k is contingent on the number of Gaussian distributions incorporated in the model. n denotes the total number of samples present in the dataset. The Akaike Information Criterion serves as another model selection criterion and is mathematically defined as follows [39]:

(3) AIC=−2⋅log−ikelihood+2k

**Figure 11:**
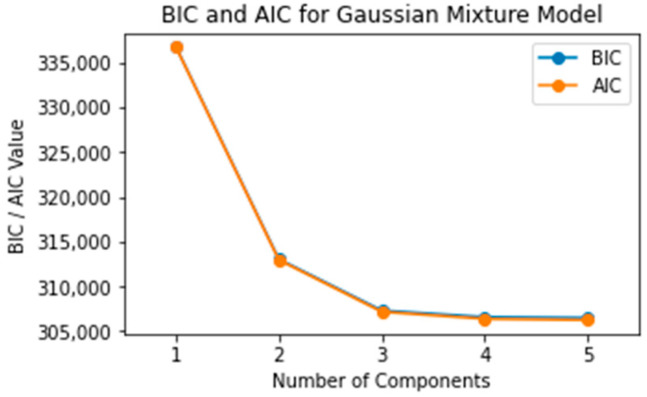
Model complexity evaluation: BIC and AIC scores as functions of Gaussian components in the GMM.

In model selection, the Bayesian Information Criterion and Akaike Information Criterion are often employed to balance goodness-of-fit with model complexity. BIC incorporates a penalty term that is more stringent compared to AIC, hence favoring simpler models when the fit is comparable. As revealed in Figure 11, both BIC and AIC values exhibit a declining trend with an increasing number of Gaussian distributions. However, the rate of this decline begins to stabilize when the model incorporates three or more Gaussian distributions. In light of this, based on the BIC and AIC criteria, the optimal number of Gaussian distributions was determined to be at least three.

In light of the observations from BIC and AIC analysis, an exploratory study was carried out to assess how the choice of the number of Gaussian distributions affects fault diagnosis outcomes. Moreover, the sensitivity of the diagnostic results to the feature engineering window size was also investigated, with the window varying from one to ten seconds. The fault diagnosis process is delineated as follows:

(1) Training the Gaussian Mixture Model.
(2) Establishing a threshold value based on the absolute log-likelihood distribution derived from stage 1.
(3) In stages 2–4, identifying data points with absolute log-likelihood values exceeding the predefined threshold as faulty.

As outlined in Table 3, a binary classification was conducted for stages 2 and 4 to differentiate between normal and abnormal conditions. In alignment with the BIC and AIC findings, Area Under the Curve (AUC) scores reached a stable level when employing three or more Gaussian components. Moreover, an incremental gain in AUC was observed with an increase in the feature engineering window size. However, extending the sliding window approach to a ten-second span did not yield any additional enhancement in the AUC metric.

For a detailed diagnosis in the unlabeled stage 3 area, anomaly detection was conducted employing two specific methods:

(1) Feature Engineering: Applying a ten-second window combined with sliding window augmentation.
(2) Gaussian Distributions: Opting for three components, a selection validated by both BIC and AIC analyses as well as expert insights in fault diagnosis.

**Figure 12:**
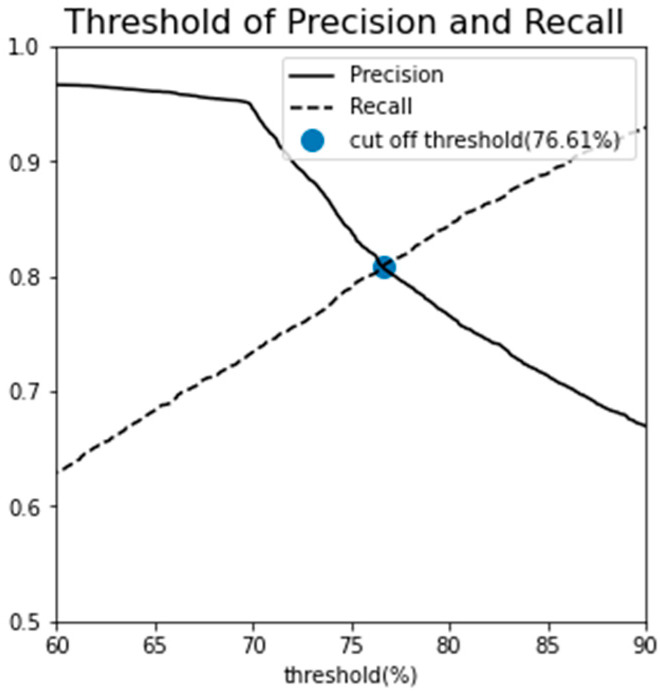
Determination of cut-off threshold and associated AUC scores Using GMM.

The number of Gaussian distributions was finalized at three, informed by both the BIC and AIC analyses as well as domain-specific expertise in fault diagnosis. The classification objective was to delineate the data into three categories: normal, semi-fault, and fault states. The choice of classification threshold is crucial as it affects the balance between precision and recall, metrics that are often inversely proportional to each other. A threshold set at 100% (indicating near-perfect recall) is prone to false negatives, potentially misclassifying abnormal data points as normal. Conversely, a lower threshold optimizes precision at the expense of recall, increasing the likelihood of mislabeling normal instances as abnormal. Given that outliers exist even within the log-likelihood distribution of stage 1 normal data, a 100% threshold would erroneously classify all outliers as normal, thereby increasing the risk of missing a critical radiator fault. To mitigate both over-detection and under-detection, an optimal threshold was sought by examining the relationship between precision and recall at various thresholds. The intersection point of these two metrics was selected as the cut-off, resulting in a threshold value of 76.61%, as shown in Figure 12.

**Figure 13:**
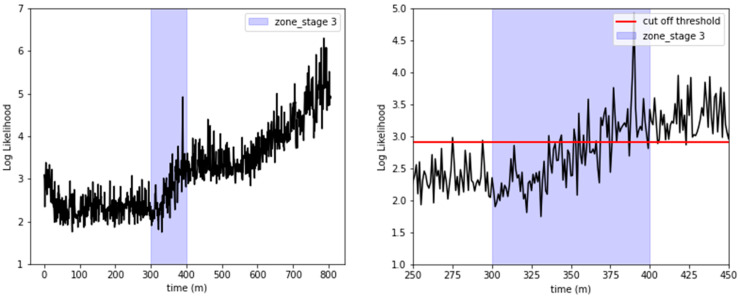
Graphical representation of log-likelihood values and anomaly detection with a defined cut-off threshold.

To examine temporal variations in the radiator state, the absolute log-likelihood values for all stages (1–4) were averaged in 60-s intervals, as illustrated in the left pane of Figure 13. During stage 1, the log-likelihood value exhibited an initial decrease before reaching a stable plateau. However, commencing at approximately 300 min, a noticeable upward trend was observed in the absolute values of the log-likelihood. This escalation crossed the established cut-off threshold at around the 400-min mark, coinciding with the visual confirmation of a coolant leak.

Moreover, a detailed examination of stage 3, showcased in the right pane of Figure 13, revealed recurrent excursions of the absolute log-likelihood values beyond the cut-off threshold, initiating at roughly 350 min. This pattern is indicative of a deteriorating radiator condition.

## 4. LSTM Autoencoder-Based Fault Diagnosis

### 4.1. Rationale for LSTM Autoencoder Selection

An autoencoder is a specialized type of neural network designed to leverage unlabeled data. Its architecture typically includes an encoder, responsible for mapping the input ($Y_{i}$) into an internal representation, and a decoder, tasked with reconstructing the output ($\hat{Y_{i}}$) from this internal representation [40]. The output, often referred to as a “reconstruction,” serves as an approximate replica of the input. The model is generally trained using Mean Squared Error (*MSE*) that measures the average squared difference between the predicted values ($\hat{Y_{i}}$) and actual values ($Y_{i}$) to quantify the reconstruction loss as follows:

(4) $$MSE=\frac{1}{n}\;\sum_{i=1}^{n}{\left(Y_{i}-\hat{Y_{i}}\right)}^{2}\;$$

**Figure 14:**
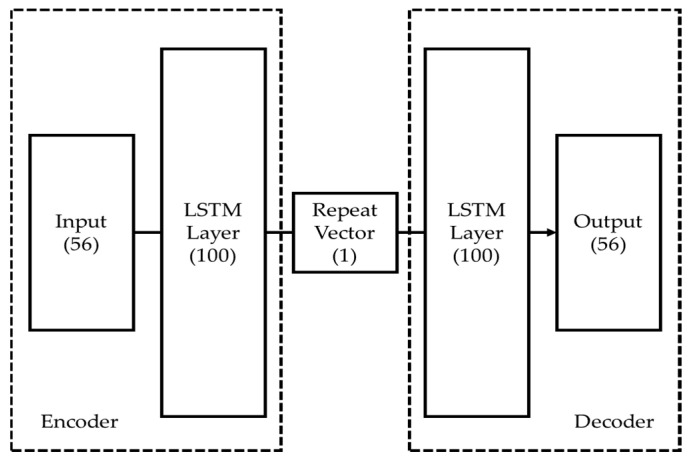
LSTM autoencoder structure that consists of two LSTM layers and a repeat vector.

LSTM networks are designed to address the vanishing gradient a limitation of traditional Recurrent Neural Networks (RNNs) [41]. This issue becomes particularly prominent in deep networks with multiple layers and nodes. Here, the initial layers might be insufficiently trained, a problem exacerbated by increasing sequence lengths. Such scenarios can lead to gradients that diminish during the backpropagation process [42,43]. Contrasting traditional RNNs, LSTMs effectively circumvent the vanishing gradient issue by incorporating both long-term and short-term state variables into the learning algorithm. This enables LSTMs to achieve successful training even over extensive data sequences, thus resolving the long-term dependency problem often found in standard RNNs [44,45]. In this study, an LSTM autoencoder was implemented, consisting of two LSTM layers specifically designed for handling time-series data, as illustrated in Figure 14. Each layer functions both as an encoder and a decoder within the overarching network structure [46]. A “repeat vector” is employed to resize the condensed latent space representation back to the initial sequence length, facilitating the decoder to reference the compressed format multiple times, which in turn enhances the fidelity of the reconstructed original input sequence.

**Figure 15:**
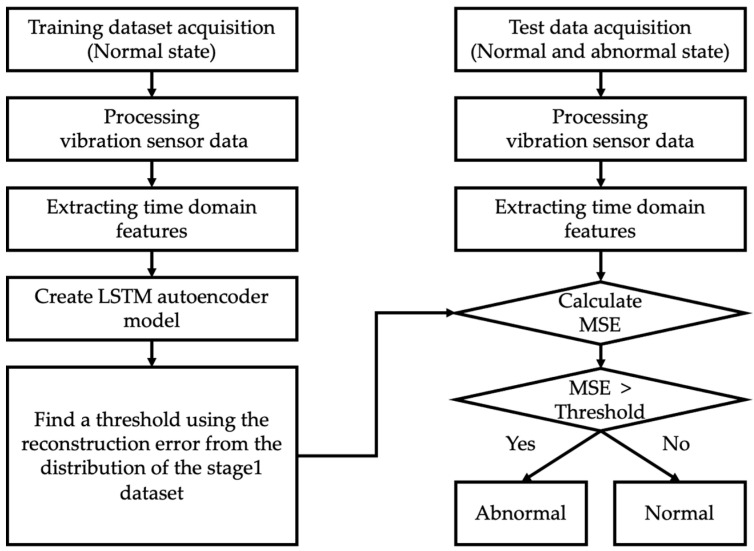
Algorithm for fault diagnosis based on the reconstruction error of the LSTM Autoencoder.

As outlined in Section 3, GMMs were trained on the comprehensive dataset covering stages 1 to 4. Thresholds for fault diagnosis were derived from the absolute log-likelihood distribution of stage 1 data. While this approach allows for effective post-hoc analysis, it might not be well-suited for proactive fault diagnosis. To address this limitation, Figure 15 introduces an LSTM autoencoder-based fault diagnosis algorithm. A previous set comprising 56 feature vectors served as the training dataset. The LSTM autoencoder was initially trained on stage 1 data, which consists exclusively of normal states. A threshold was determined from the Mean Squared Error distribution of the stage 1 training data. If the MSE for data from stages 2 to 4 falls below this threshold, the state is classified as normal; if it exceeds the threshold, the state is considered abnormal. Additionally, MSE was employed as a fault level index, providing an insightful visual metric indicative of the radiator’s deterioration trajectory.

### 4.2. Fault Diagnosis and Anomaly Detection Result Using LSTM Autoencoder

In line with the GMM analysis, the appropriate window size was examined, spanning from one to ten seconds for feature engineering. Additionally, a sliding window augmentation method was applied to the ten-second window to increase the number of data points. When evaluating the model’s performance, experiments were conducted by varying the number of nodes in the LSTM hidden layer for the Autoencoder. Furthermore, the dropout rate was adjusted to mitigate overfitting [47], and the L2 regularization parameter was modified to assess its impact on performance [48]. The experimental results, as summarized in Table 4, revealed that the AUC score for fault diagnosis exhibited a gradual improvement with an increasing window size for feature engineering. Moreover, the utilization of the sliding augmentation technique led to an enhancement in the AUC score, increasing it from 0.9728 to 0.9893. Furthermore, an increase in the number of nodes in the hidden layer also resulted in an improved AUC score. Conversely, a decline in the AUC score was observed when dropout and L2 regularization were applied.

Consistent with the GMM analysis, a comprehensive diagnosis of the unlabeled stage 3 area encompassed anomaly detection employing three methods:

(1) Feature engineering method: Employing a 10-s window alongside sliding augmentation.
(2) LSTM AE structure: Configured as 100/1/100 architecture.
(3) Dropout and L2 Regularization: Not implemented.

Following a similar analytical approach as demonstrated in Figure 12 of Section 3.2, the establishment of an appropriate MSE threshold aimed to strike a balance between over-detection and under-detection. This was achieved by identifying the intersection point through the analysis of precision and recall at various threshold values since precision and recall inherently possess a trade-off relationship. 

**Figure 16:**
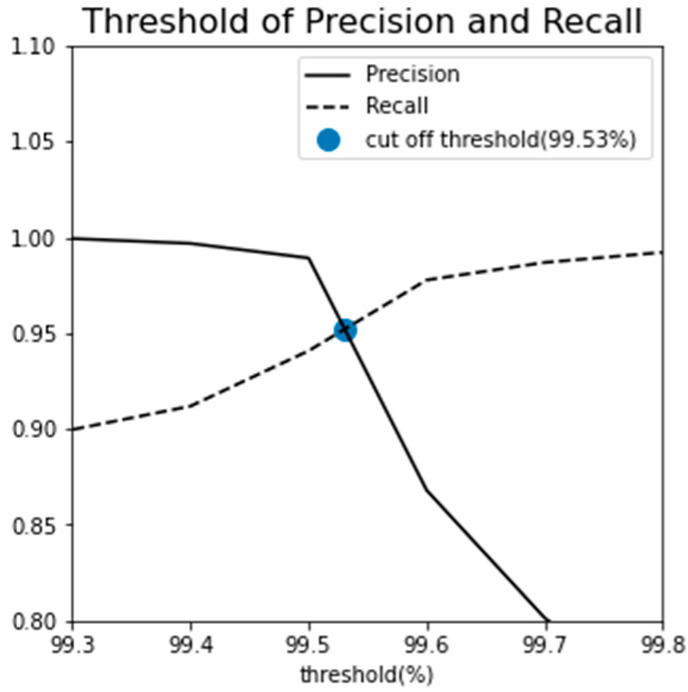
Cut-off threshold and AUC result of LSTM autoencoder.

Setting Recall to 1.00 at a 100% fault diagnostic threshold might lead to classifying faults as normal conditions. Conversely, aiming for a Precision close to 1.00 can result in normal conditions being erroneously classified as failures. Selecting the threshold at the intersection point of Precision and Recall minimizes both over-detection and under-detection. Therefore, using the intersection point as the threshold ensures that the predictive model maintains accuracy while not missing important changes in the system’s state. As shown in Figure 16, the intersection point has been confirmed to be at 99.53%. Consequently, it was determined that a threshold of approximately 99.53% was necessary for diagnosing radiator faults.

**Figure 17:**
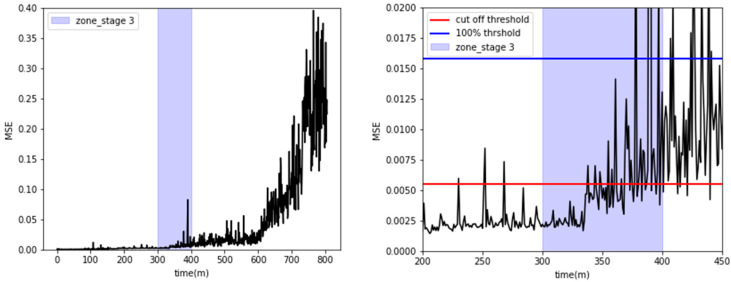
Visualization of MSE and anomaly detection with the cut-off threshold.

The MSE values for all stages 1–4 were averaged every 60 s to track changes in the radiator’s condition over time, as depicted in the left side of Figure 17. The MSE magnitude exhibited a gradual increase as time progressed. Notably, the MSE values crossed the cut-off threshold at approximately 400 min, which coincided with the visual confirmation of a coolant leak. From approximately 250 min onwards, it is evident that some spikes exceed the threshold. Acceleration sensors are sensitive to environmental conditions, and noise can be generated by external factors. The variation in sensor measurements may also impact prediction accuracy. It is inferred that the spikes exceeding the threshold occurred because the LSTM autoencoder was sensitive to the noise in the acceleration signals. Typically, equipment degrades gradually before reaching a failure state. Therefore, it is not reasonable to infer a failure based solely on a spike observed at a single point in time. Instead, it is more appropriate to continuously monitor whether the threshold is consistently exceeded to make a determination of failure or anomaly. Taking this into account, it was considered that once the MSE continuously exceeded the threshold, the system had entered a fault condition. Therefore, the transient spikes observed around 250 min were considered to be normal since they did not continuously exceed the threshold. Of course, the decision results of both GMM, as shown in Figure 12, and LSTM were overlaid to determine the presence of anomalies.

Additionally, as shown on the right side of Figure 17, during stage 3, the MSE was observed to repeatedly surpass the cut-off threshold from around the 335 to 340-min mark, indicating radiator failure. This observation closely aligned with the GMM analysis, which also pinpointed the onset of radiator issues at around 350 min.

## 5. Discussion

In this study, fault diagnosis for radiators was conducted using both GMM and LSTM autoencoder models. Initially, the radiator underwent a random durability vibration bench test to accelerate vibration-induced failure and acquire acceleration signals. The vibration data was divided into stages 1–4, and time-domain statistical features were extracted for efficient data analysis using a one- to ten-second window for feature engineering. Additionally, the feature vector was augmented by applying a sliding technique to ten-second windows.

The appropriate number of principal components was determined, and two principal components were trained on the GMM. Additionally, the GMM was employed to establish thresholds based on the absolute log-likelihood distribution of stage 1 for diagnosing faults in stages 2 and 4. The GMM model achieved a maximum AUC of 0.9009. The cut-off threshold was set at the point of intersection between precision and recall. Using this threshold, anomaly detection in stage 3 enabled the diagnosis of faults in the unlabeled state. However, it is worth noting that different distributions were observed even within the normal state of the training data. Achieving an AUC score higher than 0.9 when performing fault diagnosis using the stage 1 threshold proved challenging.

The GMM-based fault diagnosis had a limitation in that the entire dataset encompassing stages 1 to 4 was trained collectively. Consequently, GMM was not suitable for predicting faults in advance. To address this, a fault diagnosis algorithm was developed utilizing an LSTM autoencoder, which was exclusively trained on the normal state data from stage 1. This approach aimed to avoid the need for post-fault event diagnosis. Thresholds were extracted from the MSE distribution observed in stage 1, and fault diagnosis was subsequently conducted in stages 2 and 4. The experimental results demonstrated that this algorithm achieved a maximum AUC of 0.9912. Similar to the GMM, anomaly detection was performed in the unlabeled stage 3 using the cut-off point where precision and recall intersect. Furthermore, an additional analysis was carried out using the 100%-point threshold from the stage 1 MSE distribution, which closely approximates the cut-off point. Setting the threshold at 100% resulted in similar time frames for fault occurrence and alarm generation. In such scenarios, the potential for higher opportunity costs and reduced equipment productivity due to reactive maintenance could be anticipated. Conversely, adopting the cut-off threshold as the fault alarm criterion enables earlier radiator maintenance, minimizing the opportunity cost associated with equipment downtime and ensuring effective radiator maintenance.

## 6. Conclusions

This study has demonstrated an approach to enhance the accuracy of reliability life assessment in Accelerated Life Tests. The investigation focused on radiator fault diagnosis through the adjustment of thresholds using the absolute log-likelihood distribution of the GMM and the reconstruction error distribution of the LSTM autoencoder model. In the future, it is possible to achieve efficient radiator maintenance by utilizing the log-likelihood and MSE as fault level indicators and optimizing the balance between equipment lead time and opportunity cost through the establishment of appropriate radiator maintenance thresholds. Additionally, there is potential for universal application of outlier detection and fault diagnosis using the LSTM autoencoder, with training exclusively on normal state datasets. Furthermore, the fault diagnosis process can be further refined through the integration of updated fault cases and labels, existing supervised classifiers, and expert knowledge following hidden failure detection.

## Figures and Tables

**Table 1:** Results of radiator vibration tests and dataset labeling.

State	Label	Time	Observation Result
Stage 1	Normal 1	0–100 min	No coolant leakage in 100 min
Stage 2	Normal 2	100–300 min	No coolant leakage in 300 min
Stage 3	Unknown	300–400 min	Estimate a coolant leakage between 300 and 400 min
Stage 4	Abnormal	400–808 min	Coolant leakage at 400 min

**Table 2:** Data format and the specification of data acquisition system and sensors (Siemens SCADAS recorder and Isotron accelerometers).

Category	Description
Variable type	Numerical data
Sampling rate	12,800 Hz
Measurement unit	m/s^2^
Data Acquisition Equipment	Siemens SCADAS recorder with SCM-V24-II module - 24 channel input modules for tri-axial, 24 individual accelerometers - 24-bit analog-to-digital conversion with bandwidth of 23 kHz - Max sampling rate: 51.2 kHz, can be down-sampled in steps of 2 and 2.5 - 140 dB dynamic range eliminates
Sensors	Isotron accelerometer: Model 65-10 - Eight sensors were used - Manufacturer: ENDEVCO - Triaxial, but only used Y-axis - Sensitivity: 10 mV/g or 1.02 mV/(m/s^2^) - measurement range: +/− 500 g or 4905 m/s^2^ - Uncertainty estimate (95% confidence, k = 2) : +/− 1.0%, 10.0 < frequency <= 100.0 Hz : +/− 1.0%, 100.0 < frequency <= 10,000.0 Hz : +/− 2.1%, 10,000.0 < frequency <= 15,000.0 Hz
Missing data	None

**Table 3:** Classification results for stages 2 and 4 based on GMM analysis.

Window Size	The Number of Gaussian Components				
	1	2	3	4	5
1 s	0.6286	0.7018	0.7094	0.7095	0.7076
2 s	0.6182	0.7536	0.7649	0.7630	0.7609
3 s	0.6117	0.7872	0.8016	0.7971	0.7979
4 s	0.6070	0.8183	0.8306	0.8291	0.8293
5 s	0.6125	0.8384	0.8483	0.8532	0.8517
6 s	0.6141	0.8572	0.8674	0.8704	0.8717
7 s	0.6153	0.8696	0.8828	0.8822	0.8823
8 s	0.6240	0.8642	0.8833	0.8852	0.8871
9 s	0.6275	0.8724	0.8915	0.8904	0.8899
10 s	0.6226	0.8873	0.9000	0.8995	0.9009
10 s (sliding)	0.6310	0.8873	0.8998	0.8985	0.8998

**Table 4:** LSTM autoencoder-based stages 2 and 4 classification result.

Variables	Window Size	Hyperparameters			AUC
		LSTM AE Structure	Dropout Rate	L2 Regularization	
Window size	1 s	100/1/100	None	None	0.8579
	2 s	100/1/100	None	None	0.9151
	3 s	100/1/100	None	None	0.9631
	4 s	100/1/100	None	None	0.9639
	5 s	100/1/100	None	None	0.9572
	6 s	100/1/100	None	None	0.9656
	7 s	100/1/100	None	None	0.9672
	8 s	100/1/100	None	None	0.9647
	9 s	100/1/100	None	None	0.9683
	10 s	100/1/100	None	None	0.9728
	10 s (sliding)	100/1/100	None	None	0.9893
The number of nodes	10 s (sliding)	200/1/200	None	None	0.9902
	10 s (sliding)	300/1/300	None	None	0.9912
Dropout rate	10 s (sliding)	100/1/100	0.3	None	0.9868
	10 s (sliding)	100/1/100	0.5	None	0.9845
	10 s (sliding)	100/1/100	0.7	None	0.9782
L2 Regularization	10 s (sliding)	100/1/100	None	0.1	0.9729
	10 s (sliding)	100/1/100	None	0.01	0.9853
	10 s (sliding)	100/1/100	None	0.001	0.9702
Dropout rate +L2 Regularization	10 s (sliding)	100/1/100	0.5	0.01	0.9751

## Data Availability

In accordance with company security policies, the data presented in this study are available upon request from the corresponding author. The data derived from the present study are only partially available for research purposes.
